# Actions of the antihistaminergic clemastine on presymptomatic SOD1-G93A mice ameliorate ALS disease progression

**DOI:** 10.1186/s12974-016-0658-8

**Published:** 2016-08-22

**Authors:** Savina Apolloni, Paola Fabbrizio, Susanna Amadio, Cinzia Volonté

**Affiliations:** 1 Santa Lucia Foundation, IRCCS, Rome, Italy; 2 Institute of Cell Biology and Neurobiology, CNR, Via del Fosso di Fiorano, 65, 00143 Rome, Italy

**Keywords:** Neuroinflammation, Antihistamine, Primary adult microglia, Autophagy, Spinal cord

## Abstract

**Background:**

Amyotrophic lateral sclerosis (ALS) is a disease with a strong neuroinflammatory component sustained by activated microglia contributing to motoneuron death. However, how to successfully balance neuroprotective versus neurotoxic actions by the use of antinflammatory agents is still under scrutiny. We have recently shown that the antihistamine clemastine, an FDA-approved drug, can influence the M1/M2 switch occurring in SOD1-G93A ALS microglia.

**Methods:**

Here, we have chronically treated female SOD1-G93A mice with clemastine, evaluated disease progression and performed mice lumbar spinal cord analysis at symptomatic and end stage of the disease. Moreover, we have studied the mechanism of action of clemastine in primary adult spinal SOD1-G93A microglia cultures and in NSC-G93A motor neuron-like cells.

**Results:**

We found that a short treatment with clemastine (50 mg/kg) from asymptomatic (postnatal day 40) to symptomatic phase (postnatal day 120) significantly delayed disease onset and extended the survival of SOD1-G93A mice by about 10 %. Under these conditions, clemastine induced protection of motor neurons, modulation of inflammatory parameters, reduction of SOD1 protein levels and SQSTM1/p62 autophagic marker, when analysed immediately at the end of the treatment (postnatal day 120). A long treatment with clemastine (from asymptomatic until the end stage) instead failed to ameliorate ALS disease progression. At the end stage of the disease, we found that clemastine short treatment decreased microgliosis and SOD1 protein and increased LC3-II autophagic marker, while the long treatment produced opposite effects. Finally, in spinal microglia cultures from symptomatic SOD1-G93A mice clemastine activated inflammatory parameters, stimulated autophagic flux via the mTOR signalling pathway and decreased SOD1 levels. Modulation of autophagy was also demonstrated in NSC34 SOD1-G93A motor neuron-like cells.

**Conclusions:**

By gaining insights into the ameliorating actions of an antihistaminergic compound in ALS disease, our findings might represent an exploitable therapeutic approach for familial forms of ALS.

## Background

Amyotrophic lateral sclerosis (ALS) is a neurodegenerative disease characterised by selective loss of upper and lower motor neurons and with a marked multifactorial nature where genetic factors and dysregulation of crucial molecular pathways contribute to disease pathogenesis [1]. Approximately 10 % of cases can be attributed to a familial form, and one of the best characterised forms of familial ALS is caused by mutations in the gene encoding for superoxide dismutase 1 (SOD1) and occurring in about 15–20 % of familial patients. Mutated SOD1 gene can acquire both gain and loss of function deleterious mutations that modify SOD1 activity, leading to the accumulation of highly toxic intracellular aggregates and becoming one of the mechanisms sustaining ALS aetiopathogenesis together with the well-known neuroinflammation [1–4].

Emerging evidence has recently suggested the occurrence of the main degradative mechanism of misfolded proteins, autophagy [5], in the pathogenesis of ALS [6]. Altered macroautophagy is reported in the spinal cord of SOD1 mutant mice [7] and autophagy overexpression is proved in motor neurons of ALS mice by in vivo optical imaging [8]; moreover, inefficient autophagosome-lysosome fusion is reported in ALS patients [9, 10] to the point that a strategy in modulating autophagic flux may have a promise in ALS treatment [11, 12]. The p62/sequestosome 1 protein has been identified as a component of pathological protein inclusions in neurodegenerative diseases including ALS, besides being implicated in autophagy as an adaptor protein that acts as a bridge between aggregates and autophagy clearance [13]. Although autophagy constitutes a physiological mechanism to degrade proteins that is generally beneficial for cells, if uncontrolled, it may be extremely harmful for neuronal survival [6].

The histaminergic system is involved in numerous actions, comprising sensory and motor functions, and histamine-related drugs have been lately recognised to have a therapeutic role in CNS pathologies [14]. For instance, histamine H3 receptor antagonism inhibits mTOR phosphorylation and reinforces autophagy, thus protecting against ischaemic injury [15]. Latrepirdine, an antihistamine that regulates the metabolism of the amyloid-β/A4 precursor protein and autophagy, ameliorates disease pathology in an Alzheimer mouse model [16]. Moreover, latrepirdine reduces γ-synuclein protein aggregates in vivo, slowing progression of proteinopathy in γ-synuclein transgenic mice [17]. Recently, latrepirdine administered until symptomatic phase was shown to increase life span in SOD1-G93A mice by activating the autophagy-related adenosine 5′-monophosphate-activated protein kinase [18].

Clemastine (Tavegil™), an antihistamine drug originally marketed for the treatment of allergic rhinitis, is currently on phase II clinical trial for patients with relapsing forms of multiple sclerosis (www.clinicaltrial.gov), due to its recently discovered remyelinating properties [19, 20]. We have recently identified clemastine as a compound also capable of counteracting spinal cord pathology and neuroinflammatory responses in the SOD1-G93A mouse model of ALS, however not affecting mice survival, when administrated at 10 mg/kg from pre-symptomatic to the end stage of the disease [21].

Here we have administrated a higher dose of clemastine (50 mg/kg) in SOD1-G93A mice under two regimens, one up to symptoms onset and one up to the end stage of the disease. Moreover, we have dissected the actions of clemastine in SOD1-G93A lumbar spinal cord tissues, primary adult SOD1-G93A microglia and motor neuron like cultures, in order to investigate its pharmacological potential in perturbing ALS pathogenesis.

## Methods

### Reagents

Bafilomycin - A1, clemastine, wortmannin and all reagents, unless otherwise stated, were purchased from Sigma-Aldrich (Italy). Sulfobutylether-beta-cyclodextrin from Hycultec (Germany).

### Mice

Adult B6.Cg-Tg(SOD1-G93A)1Gur/J mice expressing high copy number of mutant human SOD1 with a G93A substitution (SOD1-G93A) were originally obtained from Jackson Laboratories (USA) and bred in our indoor animal facility. Transgenic hemizygous SOD1-G93A males were crossbred with C57BL/6 females, both maintained on the C57BL/6 genetic background.

Because there is gender difference in the SOD1-G93A mice response to pharmacological treatments [22], we decided to use only females, in order to avoid gender diversity. Animals were housed at constant temperature (22 ± 1 °C) and relative humidity (50 %) with a regular 12-h light cycle (light 7 am–7 pm) throughout the experiments. Food and water were freely available. When animals reached the score of 3.5 (see below), macerated food was given daily for easy access to nutrition and hydration. All animal procedures were performed according to the European Guidelines for the use of animals in research (2010/63/EU) and the requirements of Italian laws (D.L. 26/2014). The ethical procedure was approved by the Animal Welfare Office, Department of Public Health and Veterinary, Nutrition and Food Safety, General Management of Animal Care and Veterinary Drugs of the Italian Ministry of Health (protocol number 167/2013B). All efforts were made to minimise animal suffering and the number of animals necessary to produce reliable results. Transgenic progeny were genotyped by analysing tissue extracts from tail tips, as previously described [23].

### Pharmacological treatments with clemastine and analysis of disease progression

We administered clemastine at a concentration of 50 mg/kg body weight dissolved in 30 % sulfobutylether-β-cyclodextrin (SBE) for five times a week by intraperitoneal injection starting from postnatal day 40 (a time that provided promising results with a lower dose of 10 mg/kg) until either the end stage or postnatal day 120. Vehicle groups received 30 % SBE. SBE is a chemically modified cyclodextrin with a structure designed to optimise the solubility and stability of drugs. To determine disease onset and neuromuscular function, mice were subjected to grip test twice a week, starting at 70 days of age. Briefly, the following hanging wire test was used for the grip test: the mouse was placed on a wire grid (wire thickness, 2 mm) that was gently shaken to prompt the mouse to hold onto the wire and the grid was then turned upside down. The latency for the mouse to release the grid was recorded within three attempts for an arbitrary maximum of 90 s [24]. If a mouse dropped from the grid within 90 s in three consecutive trials, it was defined as symptomatic. Behavioural scores and body weights were assessed two times a week beginning at 40 days of age, in order to monitor disease progression. Behavioural score employed a rating scale from 5 (healthy without any symptom of paralysis) to 1 (fully paralysis of the hind limbs, animals predominantly lie on the side, and are not able to straighten up within 30 s, after turning them on the back) according to Apolloni et al. [21]. After reaching a score of 1, the animals were euthanized, according to the guidelines for preclinical testing and colony management [25].

### Adult spinal cord microglia cultures

Adult primary microglia from spinal cord were prepared from symptomatic, i.e. postnatal day 120 SOD1-G93A female mice. Briefly, mice were euthanized by CO2 asphyxiation and lumbar spinal cords were taken and submerged in a Petri dish containing ice-cold HBSS. After removing the meninges, the spinal cords were minced and digested with trypsin (2.5 g/L irradiated porcine trypsin and 0.2 g/L EDTA in HBSS) and incubated at 37 °C for 30 min. After dissociation and passage through 40-μm filter, cells were then plated in 35-mm poly-d-lysine coated plates. After 4 h, non-adherent cells were washed off. We obtained a population of ≥98 % microglia enriched in CD11b immunoreactivity and negative for astrocyte-GFAP marker.

### Mouse motor neuron cell line NSC34 SOD1-G93A

Construction of NSC34 cells stably transfected with mutant human SOD1-G93A DNA was described elsewhere [26]. Cells were grown in DMEM/F12 medium supplemented with 10 % FBS (Invitrogen, USA). Induction of SOD1-G93A gene was obtained by adding 1 μg/ml doxycycline to the culture medium for 48 h and verified by western blot with anti-SOD1 antibody.

### Western blot analysis

A section of lumbar spinal cord tissue was collected and homogenised in Triton X-100 containing protease inhibitor cocktail (Sigma-Aldrich) by sonication. Cell cultures were harvested in SDS Laemmli sample buffer. Separation of protein components was performed by Mini-PROTEAN® TGX™ Gels (BioRad, USA), followed by transfer onto nitrocellulose membranes (Amersham Biosciences, Italy). After saturation with 5 % non-fat dry milk, blots were probed overnight at 4 °C with the specific primary antibodies: rabbit anti-ARG1 (1:1000, Millipore, USA), rat anti-CD68 (1:500, AbD Serotech, UK), rabbit anti-CD163 (1:100, Santa Cruz Biotechnology, USA), rabbit anti-Iba1 (1:1000, Wako, USA), rabbit anti-LC3B (1:1000, Cell Signaling Technology Inc, USA), rabbit anti-mTOR and phospho-mTOR (1:1000, Cell Signaling Technology Inc), rabbit anti-nuclear factor-kappa B (NF-kB) (1:500, Cell Signaling Technology Inc), rabbit anti-P2X4 (1:500, Alomone Labs, Israel), rabbit anti-P2X7 (1:500, Alomone Labs), rabbit anti-P2Y12 (1:200, Anaspec, USA), anti-p44/42 MAPK (ERK1/2) (L34F12) mouse antibody (1:1000) and anti-phospho-p44/42 MAPK (ERK1/2) (Thr202/Tyr204) (1:1000, Cell Signaling Technology Inc.), rabbit anti-SOD1 (1:1000, Enzo Life sciences, USA), mouse anti-SQSTM1/p62 (1:1000, Abcam, USA). After incubation for 1 h with a specific HRP-conjugated secondary antibody, blots were visualised using ECL Advance Western blot detection kit (Amersham Biosciences). Signal intensity quantification was performed by Kodak Image Station analysis software. Values were normalised with mouse anti-GAPDH (1:2500, Calbiochem, USA).

### Immunofluorescence and confocal microscopy

Mice were anaesthetized by intraperitoneal injection with chloral hydrate (500 mg/kg) and were perfused intracardially with 50 ml of PBS, followed by 4 % paraformaldehyde (PFA) at pH 7.4. Immunofluorescence analysis was performed as described [21]. Spinal cord sections were incubated with mouse monoclonal anti-Neuronal Nuclei (NeuN) (1:200, Millipore). SOD1-G93A primary microglia were fixed for 20 min in 4 % PFA, permeabilized for 5 min in PBS containing 0.1 % Triton X-100. The cells were then incubated overnight at 4 °C in 1 % BSA in PBS with rabbit anti-ARG-1 (1:200), mouse anti-CD68 (1:100, Santa Cruz), rat anti-CD11b (1:200,Wako), rabbit anti-CD163 (1:30), mouse anti-glial fibrillary acidic protein (GFAP) (1:10, AbD Serotech), rabbit anti-P2Y12 (1:50), rabbit anti-SOD1(1:400), rabbit anti-LC3B (1:400) and mouse monoclonal anti-NeuN (1:200). The cultures were stained for 3 h with appropriate secondary antibodies. Nuclei were stained with 1 μg/ml Höechst 33258 for 5 min, and the cells were finally mounted and coverslipped with gel/mount anti-fading (Biomeda, USA). Immunofluorescence was analysed by means of a confocal laser scanning microscope (Zeiss, LSM700, Germany), using always identical acquisition parameters (laser intensity, photomultiplier gain, pinhole aperture, electronic zoom) for all the images. The brightness and contrast of the digital images were adjusted using the Zen software.

### Nissl staining

Mice were anaesthetized by intraperitoneal injection with chloral hydrate (500 mg/kg) and were perfused intracardially with 50 ml of PBS, followed by 4 % PFA at pH 7.4. Serial spinal cord sections (*n* = 12) from L3–L5 were randomly selected and after hydration stained with 1 % cresyl violet. Stained sections were dehydrated gradually in 50–100 % alcohol, cleared in xylene, and coverslipped with Eukitt (Sigma-Aldrich). The whole ventral horn of the spinal cord was photographed at ×20 magnification with Zeiss Axioskop 2 microscope. Polygonal-shaped neurons larger than 20 μm, with a well-defined cytoplasm with nucleus and nucleolus [27] were then counted using Neurolucida software (MBF Bioscience, USA).

### Statistical analysis

Data are presented as mean ± standard error of the means (SEM). Analysis was performed with the statistical software package MedCalc (Medcalc Software, Belgium). Mice survival was analysed with the Kaplan-Meier Graph followed by log-rank statistics. Analysis of data was performed using ANOVA. Statistical differences between groups were verified by Student’s *t* test. *p < 0.05 was considered significant.

## Results

### Clemastine short treatment prolongs survival in SOD1-G93A mice

**Fig. 1:**
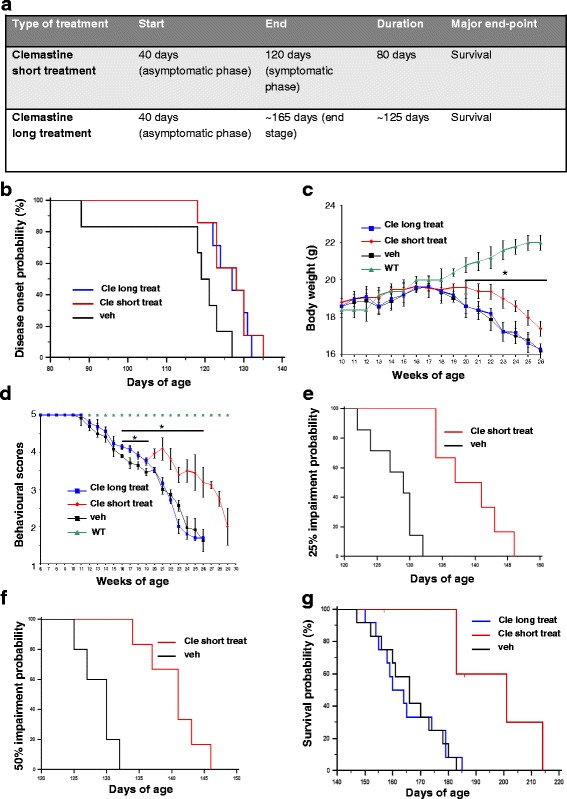
Clemastine treatment up to postnatal day 120 prolongs survival in SOD1-G93A mice. **a** Schematic representation of experimental in vivo design. **b** Short and long clemastine treatments delay disease onset of SOD1-G93A mice by 8 days (128 days for short treatment, 127 days for long treatment vs 120 days for vehicle) (*p* ≤ 0.05). **c** Body weight and **d** behavioural scores are improved in clemastine short treatment (*red line*), but not in long treatment (*blue line*), with respect to vehicle mice (*black line*) (*p ≤ 0.05). WT mice (*green line*). Short clemastine-treated mice show a significant difference in the time to reach a 25 % (**e**) and a 50 % (**f**) grip strength impairment, with respect to vehicle-treated SOD1-G93A mice (*p* < 0.05). **g** Short clemastine-treated mice (*red line*) show a significant difference in median survival, with respect to vehicle-treated SOD1-G93A mice, as shown by Kaplan–Meier survival curves (186 days for clemastine vs 170 days for vehicle; *p* < 0.05). Long clemastine-treated mice (*blue line*) show no significant differences in median survival with respect to vehicle-treated SOD1-G93A mice, as shown by Kaplan–Meier survival curves (160 days for clemastine vs 160 days for vehicle). WT group, *n* = 6 mice; vehicle- and long clemastine-treated groups, *n* = 12 mice/group; short clemastine-treated, *n* = 6 mice

In order to improve the potential of clemastine in ameliorating ALS disease progression, we performed two different treatments with a high dose of clemastine (50 mg/kg) in SOD1-G93A mice, both starting at postnatal day 40 (PND) but ending at either PND 120 (short treatment) or the end stage of the disease (long treatment) (Fig. 1a).

As shown in Fig. 1b, both clemastine short and long treatment provided a delay of 8 days in disease onset with respect to vehicle mice (128 days in short treatment, 127 days in long treatment vs 120 days in vehicle), as established by hanging grip test.

Conversely, only clemastine short treatment (red line) ameliorated body weight (Fig. 1c) and behavioural scores (Fig. 1d) until the late phases of disease. Moreover, it increased the time to reach stages of moderate deterioration, as evaluated by measuring the impairment in the hanging grip test of 25 % (128 days for vehicle vs 139 days for clemastine, Fig. 1e) and of 50 % (131 days for vehicle vs 142 days for clemastine, Fig. 1f). Finally, clemastine short treatment (red line) significantly improved survival by 16 days (~10 %) with respect to the vehicle mice (Fig. 1g). When clemastine was instead administrated in SOD1-G93A mice up to the end stage (long treatment, blue line), it failed to affect disease progression (Fig. 1c) and survival (Fig. 1g).

### Clemastine short treatment affects inflammatory markers and protects motor neurons in the lumbar spinal cord of SOD1-G93A mice at symptomatic phase

**Fig. 2:**
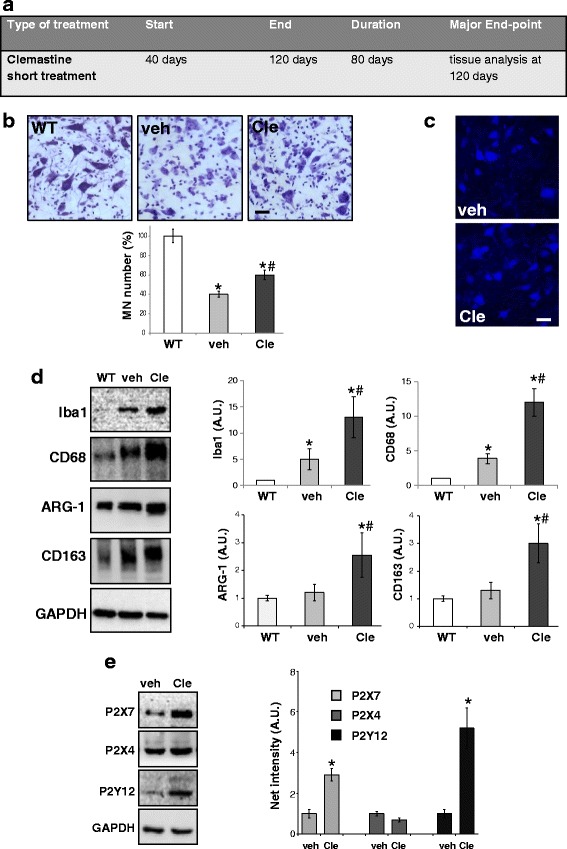
Clemastine short treatment affects the spinal cord pathology in SOD1-G93A mice at symptomatic phase of the disease. **a** Schematic representation of experimental design. **b** Spinal cord sections (L3–L5) from WT (~120 days) and vehicle- or clemastine-treated SOD1-G93A mice at PND 120 (*n* = 4/group) are stained with cresyl violet (scale bar 100 μm) and assessment of motoneuron number is performed by percentage quantification (*n* = 4/group) (*t* test referred to WT *p < 0.05 or to vehicle-SOD1-G93A, # *p* < 0.05). **c** Spinal cord sections of vehicle- or clemastine-treated SOD1-G93A mice at PND 120 were then stained with anti-NeuN (scale bar = 40 μm). **d** Equal amounts of total lumbar spinal cord lysates from WT mice (~120 days) and vehicle- or clemastine-treated SOD1-G93A mice at PND 120 (n = 3/group) are subjected to western blotting with anti-Iba1, anti-CD68, anti-ARG-1, anti-CD163 and anti-GAPDH for protein normalisation. Data represent mean ± S.E.M. Statistical significance is calculated by student’s *t* test referred to WT, * *p* < 0.05, or to vehicle SOD1-G93A mice, #*p* < 0.05. **e** Equal amounts of lumbar spinal cord lysates from vehicle- or clemastine-treated SOD1-G93A mice at PND 120 (*n* = 3/group) are subjected to western blotting with anti-P2X7, anti-P2X4, anti-P2Y12 and anti-GAPDH. Data represent mean ± S.E.M. Statistical significance was calculated by student’s *t* test, as referred to vehicle-treated SOD1-G93A mice, * *p* < 0.05

In order to investigate the beneficial effects of clemastine short treatment in SOD1-G93A mice, we analysed lumbar spinal cord tissues at PND 120 (Fig. 2a). To examine motoneuron survival, we performed Nissl staining in L3–L5 spinal cord (Fig. 2b). A significant reduction of motoneuron number occurred in vehicle SOD1-G93A mice as compared to wild type (WT), and this was in part prevented by clemastine (respect to WT mice: motor neurons surviving in vehicle = 40.3 ± 3 %, in clemastine = 64.6 ± 5.3 %, Fig. 2b). The neuroprotective effects exerted by clemastine were further confirmed by NeuN immunofluorescence (Fig. 2c).

By analysing microglia/macrophage related markers, we proved that the protein levels of Iba1 and CD68, together with arginase-1 (ARG-1) and CD163 markers, were increased in clemastine-treated SOD1-G93A respect to vehicle mice (respect to WT mice; for Iba1, vehicle-treated mice = 5 ± 2; clemastine-treated mice = 13 ± 3.9; for CD68, vehicle-treated mice = 3.8 ± 0.7; clemastine-treated mice = 12 ± 2; for ARG-1, vehicle-treated mice = 1.2 ± 0.3; clemastine-treated mice = 2.5 ± 0.8; for CD163, vehicle-treated mice = 1.3 ± 0.3; clemastine-treated mice = 3 ± 0.7, Fig. 2d).

Finally, given the recognised role of purinergic signalling in ALS neuroinflammatory processes [28], we demonstrated that the protein levels of purinergic receptors P2X7 (2.9 ± 0.3 compared to vehicle-treated mice) and P2Y12 (5.2 ± 1 compared to vehicle-treated mice) were significantly increased by clemastine, while not statistically significant effect was observed for P2X4 protein content (Fig. 2e).

### Clemastine short treatment decreases SOD1 and SQSTM1/p62 levels in SOD1-G93A mice at symptomatic phase

**Fig. 3:**
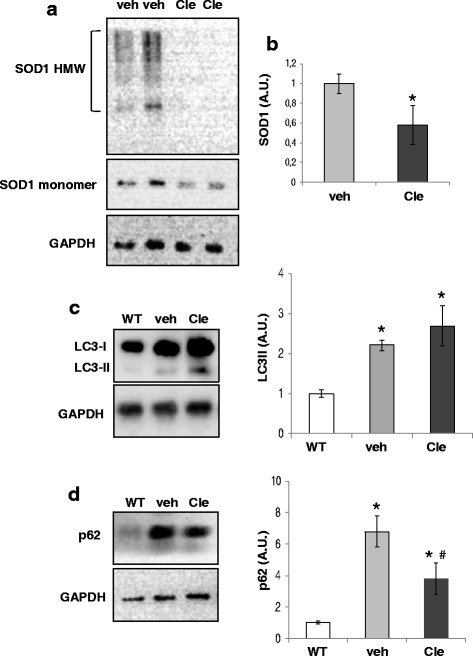
Effects of clemastine short treatment on SOD1 and SQSTM1/p62 protein levels in symptomatic SOD1-G93A mice. **a** Representative images of total lumbar spinal cord lysates from vehicle- and clemastine- treated SOD1-G93A mice at PND 120 subjected to western blotting under non-reducing conditions with anti-SOD1 and anti-GAPDH for protein normalisation. **b** Quantitative analysis of mutant SOD1 monomer band density in mice lumbar spinal cords. Data represent mean ± S.E.M. Statistical significance was calculated by student’s *t* test, as referred to vehicle-treated SOD1-G93A mice, * *p* < 0.05. **c** Equal amounts of total lumbar spinal cord lysates from WT mice (~120 days) and vehicle- or clemastine-treated SOD1-G93A mice (*n* = 4/group) are subjected to western blotting with anti LC3-I/II or in **d** with anti-SQSTM1/p62. Anti-GAPDH is used for protein normalisation. Data represent mean ± S.E.M. Statistical significance was calculated by student’s *t* test, as referred to WT, or to vehicle-treated SOD1-G93A mice, * *p* < 0.05

Strategies that limit the toxic SOD1 protein aggregation may have therapeutic potential by accelerating SOD1 degradation [11]. For this reason, we asked if clemastine might also work by reducing the levels of SOD1 protein. In clemastine-treated mice, high molecular weight SOD1 aggregates were almost undetectable (Fig. 3a) and clemastine was able to significantly decrease the level of monomeric SOD1 protein in lumbar spinal cord of SOD1-G93A mice respect to vehicle (Fig. 3b).

Since antihistamines are lately emerging as autophagy modulators, we analysed the level of the autophagic marker microtubule-associated protein 1 light chain 3-II (LC3-II), the lipidated form of LC3-I, which is augmented in SOD1-G93A respect to WT mice [29]. Confirming this result (Fig. 3c), we moreover showed that clemastine slightly increased LC3-II protein level respect to SOD1-G93A vehicle mice (respect to WT, 2.2 ± 0.1 in vehicle-treated mice and 2.7 ± 0.5 in clemastine-treated mice, *p* > 0.05, Fig. 3c). Since it is known that SQSTM1/p62 autophagy substrate accumulates in the lumbar spinal cord of ALS mice during the disease [30], we measured the level of SQSTM1/p62 protein and demonstrated that it was significantly down-regulated in clemastine-treated mice respect to vehicle (6.8 ± 1.3 in vehicle-treated mice and 3.8 ± 1.2 in clemastine-treated mice, respect to WT, Fig. 3d).

### Comparison between clemastine short and long treatment on SOD1, LC3-II, CD68 and NF-kB expression in SOD1-G93A mice spinal cord at the end stage of the disease

**Fig. 4:**
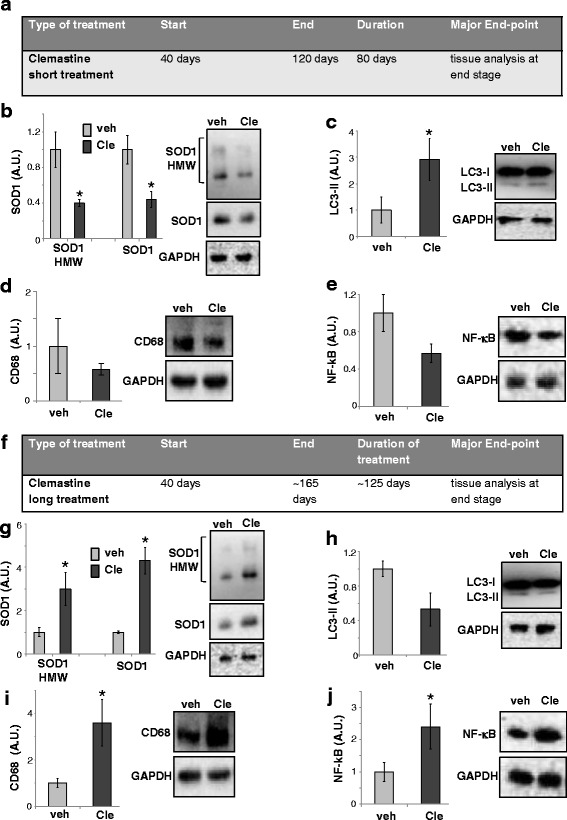
Short and long clemastine treatments differently modulate SOD1, LC3-II, CD68 and NF-kB in SOD1-G93A mice spinal cord at the end stage. **a** Schematic representation of experimental design. **b** Equal amounts of the total lumbar spinal cord lysates from vehicle and short clemastine-treated SOD1-G93A mice at the end stage (*n* = 4/group) are subjected to western blotting with anti-SOD1 under non-reducing conditions (*right panel* shows the monomer band and *left panel* shows the high molecular weight bands). Anti-GAPDH is used for protein normalisation. **c** Equal amounts of total lumbar spinal cord lysates from vehicle and short clemastine-treated SOD1-G93A mice at the end stage (*n* = 4/group) are subjected to western blotting with anti-LC3-I/II or in **d** with anti-CD68 or in **e** with anti-NF-kB. Anti-GAPDH is used for protein normalisation. Data represent mean ± S.E.M. Statistical significance is calculated by student’s *t* test referred to vehicle SOD1-G93A mice, * *p* < 0.05. **f** Schematic representation of experimental design. **g** Equal amounts of total lumbar spinal cord lysates from vehicle- and long clemastine-treated SOD1-G93A mice at the end stage (*n* = 4/group) are subjected to western blotting with anti-SOD1 under non-reducing conditions (*right panel* shows the monomer band and *left panel* shows the high molecular weight bands). **h** Equal amounts of total lumbar spinal cord lysates from vehicle- and long clemastine-treated SOD1-G93A mice at the end stage (*n* = 4/group) are subjected to western blotting with anti-LC3-I/II or in **i** with anti-CD68 or in **j** with anti-NF-kB. Anti-GAPDH is used for protein normalisation. Data represent mean ± S.E.M. Statistical significance is calculated by student’s *t* test referred to vehicle SOD1-G93A mice, * *p* < 0.05

In order to investigate if the effects of clemastine short treatment were maintained up to late phases of ALS disease, we finally analysed lumbar spinal cord tissues at the end stage of the disease (Fig. 4a). Clemastine short treatment markedly diminished SOD1 levels with respect to vehicle-treated mice (for SOD1 monomer, 1 ± 0.2 in vehicle vs 0.4 ± 0.04 in clemastine, for SOD1 high molecular weight bands 1 ± 0.1 in vehicle vs 0.4 ± 0.1 in clemastine, Fig. 4b). Concomitantly, the level of LC3-II resulted significantly increased in the same spinal cord tissue (1 ± 0.5 in vehicle vs 2.91 ± 0.8 in clemastine, Fig. 4c). Moreover, clemastine decreased CD68 protein level with respect to vehicle group, although not significantly (1 ± 0.5 in vehicle vs 0.6 ± 0.1 in clemastine, Fig. 4d, *p* > 0.05). Since a master regulator of inflammation, NF-kB that triggers microglia-induced motoneuron death in ALS, is known to be up-regulated in SOD1-G93A mice spinal cord [31], we next analysed its level in clemastine-treated mice. As shown in Fig. 4e, clemastine decreased NF-kB protein expression with respect to vehicle-treated mice (1 ± 0.2 in vehicle vs 0.57 ± 0.1 in clemastine, *p* > 0.05).

Finally, to dissect the differences occurring between clemastine short and long treatment, we next analysed the lumbar spinal cord of clemastine long-treated mice at the end stage of the disease (Fig. 4f). We demonstrated that clemastine long treatment significantly augmented SOD1 protein levels compared to vehicle (1 ± 0.2 in vehicle vs 3 ± 0.7 in clemastine, for SOD1 monomer; 1 ± 0.1 in vehicle vs 4.3 ± 0.6 in clemastine for SOD1 high molecular weight bands, Fig. 4g), while LC3-II levels were not up-regulated in the clemastine group (1 ± 0.1 in vehicle vs 0.6 ± 0.2 in clemastine, *p* > 0.05, Fig. 4h), differently from what observed with clemastine short treatment. Moreover, clemastine significantly up-regulated CD68 (1 ± 0.2 in vehicle vs 3.6 ± 0.4 in clemastine, Fig. 4i) and NF-kB (1 ± 0.3 in vehicle vs 2.4 ± 0.7 in clemastine, Fig. 4j) with respect to vehicle-treated mice.

### In primary spinal microglia from symptomatic SOD1-G93A mice, clemastine modulates inflammation and induces autophagy

**Fig. 5:**
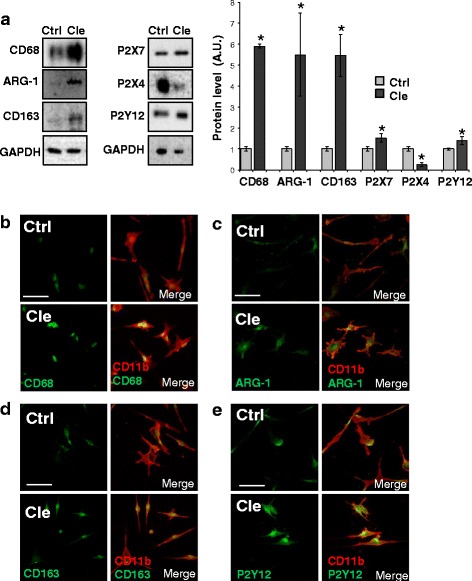
Clemastine modulates the activation pattern of SOD1-G93A spinal microglia cultures from symptomatic mice. Spinal primary microglia from SOD1-G93A mice at PND 120 are exposed to clemastine 30 μM for 6 h. **a** Equal amounts of total lysates are subjected to western blotting with anti-CD68, anti-ARG-1, anti-CD163, anti-P2X7, anti-P2X4, anti-P2Y12 and anti-GAPDH for protein normalisation. Data represent mean ± S.E.M. of *n* = 3 independent experiments. Statistical significance is calculated by student’s *t* test referred to untreated cells (*Ctrl*), * *p* < 0.05. Cells are then analysed by means of a confocal microscope (scale bar 20 μm) after staining with anti-CD11b (*red*) and in **b** with anti-CD68 (*green*), in **c** with anti-ARG-1 (*green*), in **d** with anti-CD163 (*green*) and in **e** with anti-P2Y12 (*green*)

Since inflammatory configuration of ALS microglia is regionally and temporally specific [32], in order to dissect the clemastine actions mediated by microglia, we used spinal cord microglia purified from SOD1-G93A mice at PND 120. We proved that 30-μM clemastine for 6 h modulated inflammatory features by increasing the protein expression of CD68 (5.9 ± 0.1 fold vs control) and M2-like markers arginase-1 (5.5 ± 2 fold vs control) and CD163 (6.8 ± 1 fold vs control), as well as P2X7 (1.5 ± 0.2 fold vs control) and P2Y12 (1.4 ± 0.2 fold vs control), while decreased P2X4 levels by about 70 % compared to unstimulated microglia (Fig. 5a). By double immunofluorescence and confocal analysis with anti-CD11b (microglia marker, red) and respectively anti-CD68 (green), anti-ARG-1 (green), anti-CD163 (green) or anti-P2Y12 (green) antibodies (Fig. 5b-e), we finally confirmed the up-regulation of CD68, arginase-1, CD163 and P2Y12 levels.

**Fig. 6:**
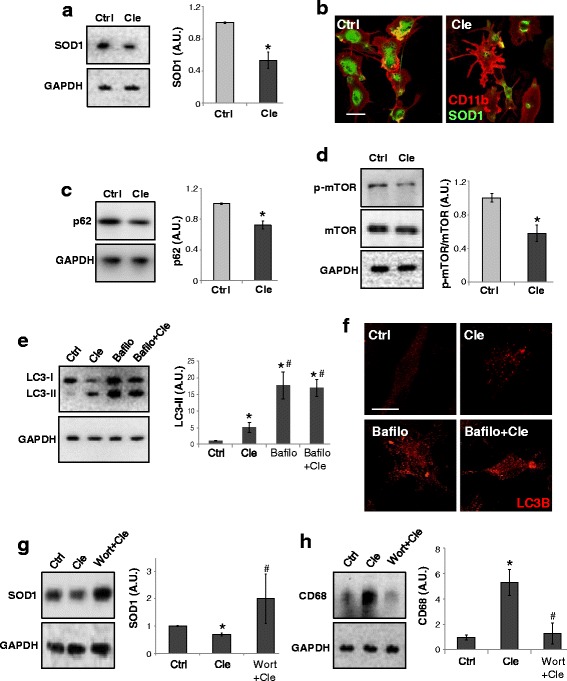
Clemastine decreases SOD1 and stimulates autophagy via mTOR pathway in SOD1-G93A spinal microglia cultures. Spinal primary microglia from SOD1-G93A mice at PND 120 are exposed to clemastine 30 μM for 6 h. **a** Equal amounts of treated microglia lysates are subjected to western blotting with anti-SOD1 and anti-GAPDH. **b** Cells are analysed by means of a confocal microscope after staining with anti-CD11b (*red*) and anti-SOD1 (*green*) (scale bar 20 μm). **c** Equal amounts of treated microglia lysates are subjected to western blotting with anti-SQSTM1/p62 or in **d** with anti-mTOR and anti-p-mTOR. Anti-GAPDH was used for protein normalisation. **e** SOD1-G93A microglia are exposed to clemastine 30 μM and/or bafilomycin-A1 25nM for 6 h, and equal amounts of total lysates are subjected to western blotting with anti-LC3-I/II and anti-GAPDH for protein normalisation. **f** Cells are also analysed by means of a confocal microscope after staining with anti-LC3-I/II (*red*) (scale bar 20 μm). Total lysates of SOD1-G93A microglia exposed to clemastine and/or wortmannin 100 nM are subjected to western blotting with anti-SOD1 (**g**) or anti-CD68 (**h**). Anti-GAPDH is used for protein normalisation. Data represent mean ± S.E.M. of *n* = 3 independent experiments. Statistical significance is calculated by student’s *t* test referred to untreated cells (Ctrl), * *p* < 0.05 or to clemastine-treated cells, ^#^ *p* < 0.05

We next studied the effect of clemastine on SOD1 level by proving that clemastine at 30 μM for 6 h reduced SOD1 protein of about 50 % respect to control SOD1-G93A microglia (Fig. 6a). By double immunofluorescence with CD11b (red) and SOD1 (green) antibodies, we demonstrated a reduced SOD1 staining intensity in clemastine-treated microglia compared to control (Fig. 6b).

By analysing autophagy marker, we then proved that the level of SQSTM1/p62 was significantly reduced by clemastine of about 30 % with respect to control cells (Fig. 6c). We next examined the signalling pathway of mTOR, a key regulator of autophagy that can be monitored by the levels of phosphorylated-mTOR (p-mTOR) [33]. We observed a significant reduction of p-mTOR in clemastine-treated cells with respect to control, suggesting enhanced autophagic activity in SOD1-G93A microglia via inhibition of the mTOR signalling pathway (Fig. 6d).

A correct way to define if a treatment augments a true autophagic flux (instead of non-productive late stage autophagy) is to determine LC3-I to LC3-II conversion in the presence of the vacuolar ATPase inhibitor bafilomycin-A1 [33]. Indeed, we demonstrated that clemastine per se increases LC3-II by 5 ± 1.6-fold respect to control cells, while the addition of bafilomycin-A1 further increased LC3-II by 16.9 ± 2.5-fold, consistently with an accelerated true autophagic flux (Fig. 6e). This was also demonstrated by immunofluorescence with anti-LC3B antibody (red), where LC3B-positive dots were indeed augmented in SOD1-G93A microglia in the presence of bafilomycin A-1 and clemastine respect to clemastine alone (Fig. 6f).

To determine if the decrease in mutant SOD1 by clemastine could be due to enhanced autophagy, we treated microglia with the phosphoinositide 3-kinase inhibitor wortmannin, known to block an early stage of autophagy [33]. As shown in Fig. 6g, in the presence of wortmannin, clemastine failed to decrease SOD1 levels. Finally, since increased autophagy can contribute to microglia activation [34], we measured CD68 protein expression in SOD1-G93A microglia pre-treated with wortmannin. We found that wortmannin reversed the up-regulation of CD68 induced by clemastine (Fig. 6h).

### Clemastine treatment affects autophagy in NSC34 SOD1-G93A cultures

**Fig. 7:**
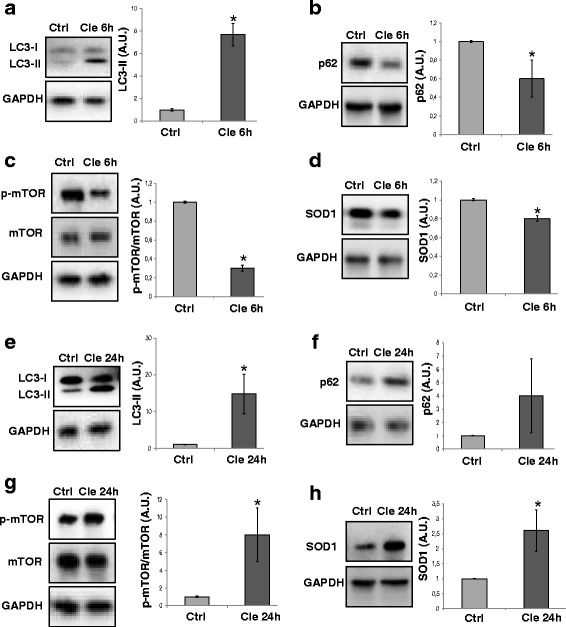
Autophagy is modulated by clemastine in a time-dependent manner in NSC34 SOD1-G93A motor neuron cells. NSC34 SOD1-G93A motor neurons are exposed to clemastine 30 μM for 6 h. Equal amounts of treated cells lysates are subjected to western blotting with anti-LC3I/II (**a**), anti-SQSTM1/p62 (**b**), anti-mTOR and anti-p-mTOR (**c**) or anti-SOD1 (**d**). Anti-GAPDH is used for protein normalisation. NSC34 SOD1-G93A motor neurons are exposed to clemastine 30 μM for 24 h. Equal amounts of treated cells lysates are subjected to western blotting with anti-LC3I/II (**e**), anti-SQSTM1/p62 (**f**), anti-mTOR and anti-p-mTOR (**g**) or anti-SOD1 (**h**). Anti-GAPDH was used for protein normalisation. Data represent mean ± S.E.M. of *n* = 3 independent experiments. Statistical significance is calculated by student’s *t* test referred to untreated cells (*Ctrl*), * *p* < 0.05

Finally, we investigated the in vitro effects of clemastine on autophagy and SOD1 levels also in a motoneuron-like cell line. As shown in Fig. 7, in NSC34 SOD1-G93A cultures, 30-μM clemastine after 6 h remarkably increased LC3II level of about eightfold with respect to control cells (Fig. 7a), concomitantly with a reduction of about 40 % in p62 protein content (Fig. 7b). We also observed a significant decrease of p-mTOR in cells treated with clemastine, whereas the total mTOR remained relatively constant (Fig. 7c). Moreover, in NSC34 SOD1-G93A cultures, clemastine significantly reduced mutant SOD1 protein, with respect to unstimulated cells (Fig. 7d). Interestingly, after a 24-h treatment with clemastine, we observed a hyper accumulation of LC3II, p62 and p-mTOR expression in NSC34 SOD1-G93A cells (Fig. 7e–g). This is consistent with an inhibition of autophagy, as described for other histamine related compounds when employed with long incubation times [35, 36]. As shown by western blot, this arrest of autophagy occurred concomitantly with an increase in SOD1 level induced by clemastine after 24 h (Fig. 7h).

## Discussion

A synergy of different pathological mechanisms such as motoneuron degeneration, microglia switch from neuroprotective M2 to deleterious M1 phenotype [37], pathological protein aggregation [38] and aberrant autophagy [39] characterise the symptomatic phase of ALS and lead to a drastic acceleration of the disease. The major finding of this work is that a treatment in SOD1-G93A mice with the antihistaminergic drug clemastine in presymptomatic phase (short treatment) affects both neuroinflammatory parameters (Iba1, CD68, arginase1, CD163, P2X7 and P2Y12) and autophagic markers (LC3-II and SQSTM1/p62), moreover decreases SOD1 protein levels and improves motoneuron survival. We thus suppose that modulation of these parameters during the presymptomatic stage of the disease might contribute to the clemastine-induced delay of ALS progression and improvement of pathological features until the end stage.

Conversely, we have also demonstrated that a clemastine treatment up to the end stage of the disease fails in ameliorating disease progression and instead increases microgliosis, NF-kB and SOD1 protein levels in the late phases of the disease. These differences in phenotypic outcomes could be explained by clemastine acting on molecular mechanisms that possess complex and dual roles in the pathogenesis of ALS, such as neuroinflammation and autophagy [37, 40, 41]. While the mild activation of these parameters by clemastine short treatment could protect motoneuron and improve mice survival, a persistent stimulation of these same pathways could be deleterious during the late phases of disease progression. If we consider that the early stage of ALS is characterised by neuroinflammation exhibiting neuroprotective M2 functions that are lost and converted into M1 toxic actions when disease progression accelerates in SOD1-G93A mice (approximately around 16–18 weeks) [37, 41], the efficacy of the short treatment could be explained by having clemastine sustained neuroinflammation only when it is protective for motor neurons. In addition, clemastine in the early phase prompts immune cells to acquire an anti-inflammatory phenotype during the late phase of the disease, as shown by the decreased expression of the proinflammatory factor NF-kB, well known to contribute to immune-mediated motoneuron death in ALS [31]. Thus, a tight balance in modulating pathological hallmarks of ALS has to be achieved by clemastine for ameliorating disease progression. This is consistent with results obtained on neuroinflammation and autophagy in ALS, whose modulators can have either beneficial or detrimental effects [30, 42].

Targeting autophagy in ALS mice has indeed demonstrated conflicting results since beneficial effects were reached with both activators as well as inhibitors. For instance, progesterone or trehalose have shown therapeutic efficacy in ALS models by activating autophagy but, on the other hand, a recent paper has demonstrated a remarkable increase in mice life span by down-regulating autophagy in motor neurons with *n*-butylidenephthalide [43]. In addition, rapamycin and lithium both enhancing autophagy have failed to ameliorate ALS disease [22, 30]; hence, the precise role of autophagy in ALS is still to be resolved. However, in the case of clemastine, we have worked within a different experimental paradigm, by having interrupted the treatment at the disease symptoms and shown that clemastine is then effective only when administered in the early-stage of the disease (short treatment) where, in addition to increase autophagy, it has beneficial immune-related actions (M2-like). Thus, we hypothesise that overstimulation of these same pathways during the long clemastine treatment when the disease accelerates might conversely activate deleterious M1-like immune responses and arrest autophagy.

The stimulation of autophagy by clemastine is consistent with the role that antihistamines H1 play in autophagy [44, 45], and with the stimulation of autophagy by antihistaminergic drugs in ALS [18], in prion-infected mice [46], in Alzheimer’s disease [16, 47] and in cerebral ischemia [15], thus suggesting that the efficacy of clemastine against ALS might also occur through the autophagic process.

In order to elucidate the clemastine-mediated actions, we have taken advantage of spinal cord primary microglia from SOD1-G93A symptomatic mice. It is known that purinergic signalling has a role in microglia [48]. In particular, P2X7 is involved in microglia-dependent neuroprotection [49], P2X4 is a microglia death receptor [50], and the P2Y12 is known to be enhanced in microglia under alternatively activated M2 conditions [51]. We have shown here that besides activating and polarising adult microglia towards a M2-like phenotype, clemastine also increases P2X7 and P2Y12 receptors, while decreasing P2X4 expression, further suggesting that clemastine prompts SOD1-G93A spinal microglia to acquire a protective state.

Interplay between inflammatory responses and autophagy in microglia is a common feature of CNS pathologies. Recently, it was reported that autophagy might influence inflammation and activation of microglia, as well as inflammation might promote or inhibit the process of autophagy [52]. In this context, our work has demonstrated that clemastine can activate autophagy in SOD1-G93A primary microglia, by increasing LC3-II and decreasing SQSTM1/p62 and p-mTOR markers, and that microglia activation by clemastine might depend, at least in part, by autophagy. This in vitro modulation of autophagy thus sustains a microglia-mediated effect of clemastine occurring also in vivo, in addition to the concomitant participation of motor neurons to the autophagic process. Our results have indeed shown that clemastine modulates LC3-II, SQSTM1/p62 and mTOR in the NSC34 SOD1-G93A motoneuron cells in a time-dependent manner, in parallel with the different effects exerted by short and long treatment in ALS mice, and thus sustaining at least in part our hypothesis.

## Conclusions

In conclusion, by analysing two pharmacological treatments with clemastine in SOD1-G93A mice, one up to symptomatic phase and the other up to the end stage of the disease, we have gained insights into the ameliorating actions of an antihistaminergic compound in ALS progression and repositioned clemastine from a drug for allergic rhinitis to a potential pharmacological compound for ALS. The beneficial effects on disease progression and survival found for the short clemastine treatment are in accordance with previous results obtained for instance with latrepirdine in SOD1-G93A mice by two different groups, showing that mice survival was improved only when the treatment was performed in the presymptomatic phase and not in the symptomatic phase [18, 53]. By analysing the implicated mechanisms, we have shown that proteostasis regulation and neuroinflammation are key targets of clemastine action. Because retinoids have been shown to significantly extend SOD1-G93A mice survival by modulating these same parameters [27, 54], and current opinion now suggests that mixed approaches combining drugs with synergic effects might be therapeutically useful, further treatments might consider the use of retinoids together with clemastine. In addition, given the sex-dependent neuroprotective effects exerted by drugs in SOD1 models [22], it will be crucial to test clemastine in male mice and, finally, to perform dose-dependent trials in order to prove the clemastine activity in affecting ALS pathogenesis.

Given the growing interest in studying presymptomatic ALS and the identification of always more genes conferring high risk for the disease [55], clemastine might then represent an exploitable therapeutic approach at least for the familial forms of ALS.

## Abbreviations

ALS, amyotrophic lateral sclerosis; H1R, histamine 1 receptor; LC3, microtubule-associated protein 1 light chain 3; NF-kB, nuclear factor-kappa B; PFA, paraformaldehyde; PND, postnatal day; SBE, sulfobutylether-β-cyclodextrin; SOD1, superoxide dismutase 1; WT, wild-type
